# Dose T2-weighted short TI inversion recovery images on cardiac magnetic resonance reflect disease activity in cardiac involvement of sarcoidosis patients?

**DOI:** 10.1186/1532-429X-14-S1-P197

**Published:** 2012-02-01

**Authors:** Yufuko Kasai, Arata Nomura, Takatomo Nakajima, Hisako Omori, Fumiko Kimura, Shuji Sakai, Nobuhisa Hagiwara

**Affiliations:** 1 Cardiology, Tokyo Women's Medical University, Tokyo, Japan; 2 Department of Diagnostic Radiology, Saitama Medical University, International Medical Center, Saitama, Japan; 3 Diagnostic Imaging and Nuclear Medicine, Tokyo Women's Medical University, Tokyo, Japan; 4 Cardiology, Seirei Hamamatsu General Hospital, Shizuoka, Japan

## Background

Cardiac involvement of sarcoidosis (CIS) in progressed and advanced stage increases cardiac events and mortality of systemic sarcoidosis, so early diagnosis and determination of disease activity are needed for treating in CIS. However, evaluating disease activity of CIS remains a major challenge because no single diagnostic test has yet been established with a high accuracy. High signal intensity of T2-weighted short TI inversion recovery images (T2-STIR) on cardiac magnetic resonance (CMR) might be useful technique for investigating myocardial edematous tissue caused by inflammation.We investigated the relationship between high signal intensity of T2-STIR and disease activity in patients with CIS.

## Methods

38 patients (male/female = 4/34, age 30-78 years) with CIS or suspect of CIS underwent T2-STIR on CMR. We defined CIS activity as follows; worsening cardiac symptoms, abnormal results of serum tests and other imaging findings.

## Results

Eleven of 38 patients (28%) had high signal intensity of T2-STIR. In these patients with high intensity of T2-STIR, 8 patients (73%) confirmed CIS activity, and 3 patients (27%) did not. In active CIS patients, 4 patients were treated with corticosteroid, then 3 patients improved cardiac symptoms or disappeared high signal intensity of T2-STIR. In the rest of all 4 patients without corticosteroid therapy showed worsening CIS.

## Conclusions

High signal intensity of T2-STIR reflects disease activity in patient with CIS.

## Funding

Nothing.

